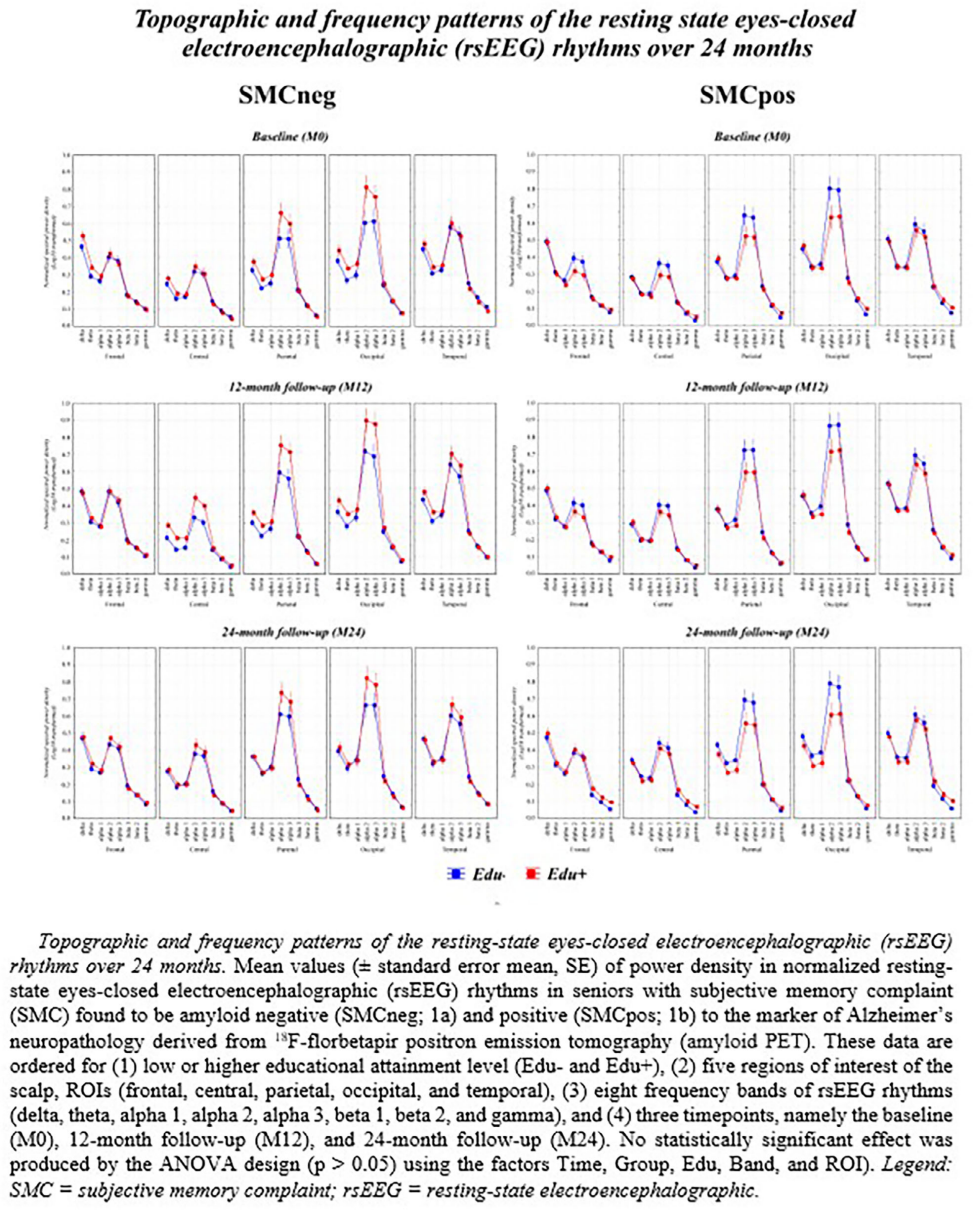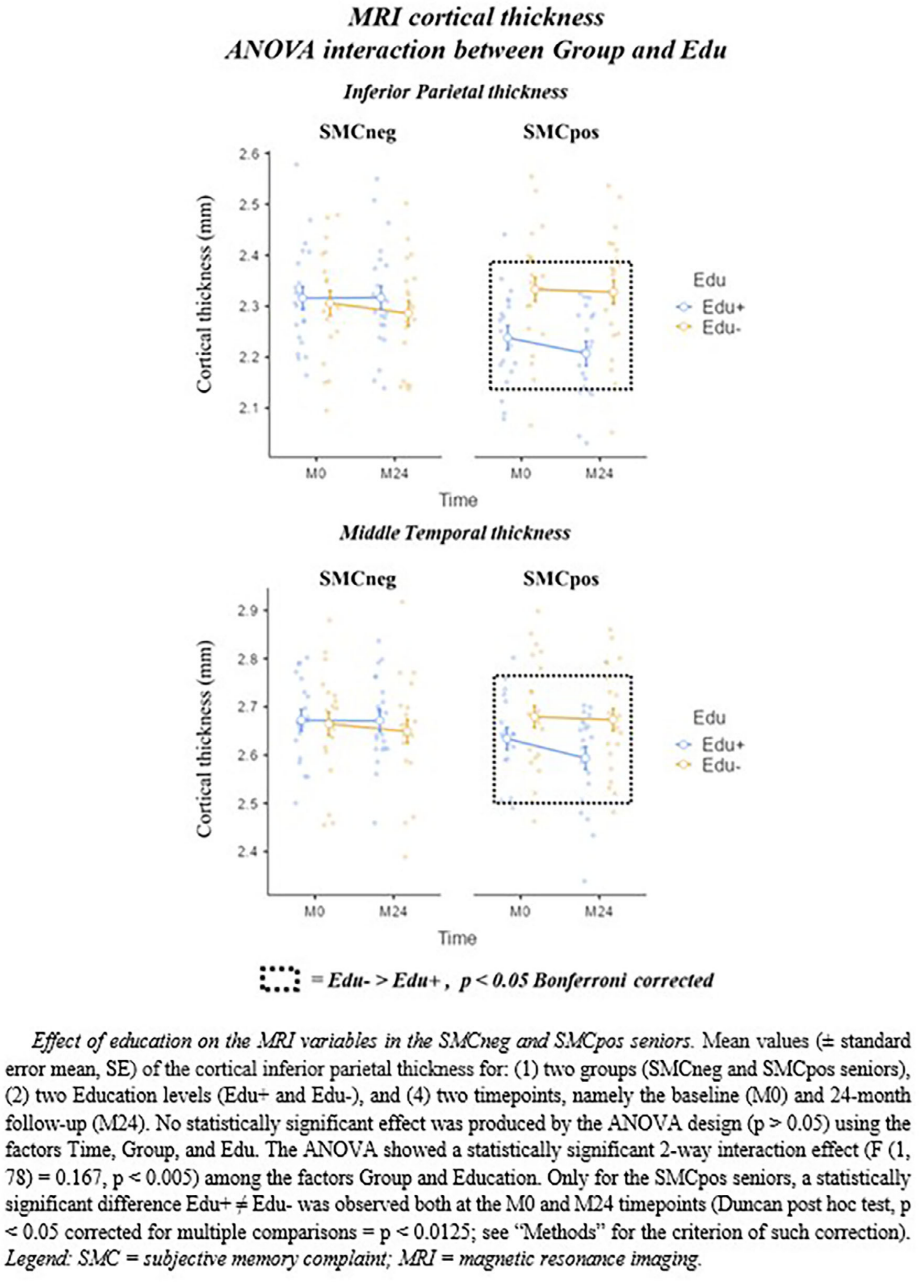# Older adults with subjective memory complaints and brain amyloidosis show stable electroencephalographic rhythms, cortical structure, and cognitive performances over 2 years

**DOI:** 10.1002/alz70856_106502

**Published:** 2026-01-07

**Authors:** Susanna Lopez, Harald Hampel, Claudio Del Percio, Giuseppe Noce, Roberta Lizio, Stefan J. Teipel, Martin Dyrba, Andrea Vergallo, Raffaele Ferri, Matteo Pardini, Claudio Babiloni

**Affiliations:** ^1^ Sapienza University of Rome, Rome, Italy; ^2^ Sorbonne University, GRC n°21, Alzheimer Precision Medicine (APM), AP‐HP, Pitié‐Salpêtrière Hospital, Boulevard de L'hôpital, F‐75013, Paris, France; ^3^ IRCCS Synlab SDN, Naples, Italy; ^4^ Oasi Research Institute – IRCCS, Troina, Italy, Troina, Italy, Italy; ^5^ Department of Psychosomatic Medicine, Rostock University Medical Center, Rostock, Germany; ^6^ German Center for Neurodegenerative Diseases (DZNE), Rostock, Germany; ^7^ Sorbonne University, GRC n° 21, Alzheimer Precision Medicine (APM), AP‐HP, Pitié‐Salpêtrière Hospital, Boulevard de l'hôpital, F‐75013, Paris, France; ^8^ Oasi Research Institute ‐ IRCCS, Troina, Italy; ^9^ DINOGMI, Università degli studi di Genova, Genova, Italy; ^10^ San Raffaele Cassino, Cassino, Italy

## Abstract

**Background:**

It is well‐known that in patients with Alzheimer's disease (AD) and high education attainment, cognitive performance is typically better than expected based on the burden of brain neuropathology and neurodegeneration (Stern et al., 2018; doi: 10.1016/j.neuroimage.2018.05.033). This resilience of the cognitive status was attributed to a sort of ***cognitive reserve*** (CR) accumulated by persons with high education attainment, which predicts a life with engaging job, intellectual, and social demands (Arenaza‐Urquijo et al. 2015; doi: 10.3389/fnagi.2015.00134; Stern et al. 2018). Previous resting‐state eyes‐closed electroencephalographic (rsEEG) studies showed that alpha rhythms in posterior visual and visuospatial areas are related to CR in healthy adults, subjective memory complaints (SMC) seniors, and patients with mild cognitive impairment due to AD (ADMCI).

**Method:**

In the present exploratory study, we used the database of the INSIGHT cohort (Dubois et al., 2018; doi: 10.1016/S1474‐4422(18)30029‐2), we investigated whether older adults with subjective memory complaints (SMC) and brain amyloid‐β accumulation may exhibit clinical progression over 2 years as a function of educational attainment (a proxy of cognitive reserve).

**Result:**

SMCneg with high educational attainment (Edu+) participants showed greater posterior rsEEG alpha rhythms compared to SMCneg with low educational attainment (Edu‐) participants. In contrast, SMCpos Edu+ participants exhibited reduced posterior rsEEG alpha rhythms and parietal cortical thickness compared to SMCpos Edu‐ participants. No EEG (Figure 1) or MRI (Figure 2) marker significantly changed over the 2‐year follow‐up period

**Conclusion:**

These findings suggest that a substantially longer time interval than 2 years should be assessed to evaluate the Alzheimer's disease progression and biomarker‐guided targeted therapies in presymptomatic SMCpos adults.